# A Comparative Study between A Protein Based Amorphous Formulation and Other Dissolution Rate Enhancing Approaches: A Case Study with Rifaximin

**DOI:** 10.3390/pharmaceutics15010126

**Published:** 2022-12-30

**Authors:** Xuezhi Zhuo, Maud Margrethe Brekstad Kjellin, Zarah Schaal, Tengyu Zhang, Korbinian Löbmann, Donglei Leng

**Affiliations:** 1 Department of Pharmacy, Faculty of Health and Medical Sciences, University of Copenhagen, Universitetsparken 2, DK-2100 Copenhagen, Denmark; 2 Zerion Pharma A/S, Blokken 11, DK-3460 Birkerød, Denmark

**Keywords:** amorphous solid dispersion, β-lactoglobulin, polymer, dissolution, nanocrystal

## Abstract

Amorphous solid dispersions (ASDs) based on proteins as co-formers have previously shown promising potential to improve the solubility and bioavailability of poorly water-soluble drugs. In particular, whey proteins have shown to be promising co-formers and amorphous stabilizers in ASD formulations, including at high drug loading. In this study, the feasibility of the whey protein β-lactoglobulin (BLG) as a co-former in ASDs was compared to the more traditional ASD co-formers based on synthetic polymers (hydroxypropyl methylcellulose acetate succinate and Eudragit^®^ L) as well as to a nanocrystalline formulation. The poorly water-soluble drug rifaximin (RFX) was chosen as the model drug. All drug/co-former formulations were prepared as fully amorphous ASDs by spray drying at 50% (*w*/*w*) drug loading. The BLG-based ASD had the highest glass transition temperature and showed a faster dissolution rate and higher drug solubility in three release media with different pH values (1.2, 4.5, and 6.5) compared to the polymer-based ASDs and the nanocrystalline RFX. In conclusion, BLG is a promising co-former and amorphous stabilizer of RFX in ASD formulations, superior to the selected polymer-based ASD systems or the nanocrystalline formulation.

## 1. Introduction

Poor aqueous solubility poses one of the most challenging limitations in modern drug development. About 90% of developmental drugs in pharmaceutical pipelines are poorly soluble, resulting in low or variable bioavailability when given orally [1]. Amongst the most frequently applied approaches to overcome poor bioavailability are particle size reduction and the use of the amorphous form of the drug. The former usually aims at preparing a crystalline product with reduced particle size, e.g., in the nanometer range, to obtain a faster dissolution of the drug due to the increased surface area [2]. The latter takes advantage of the higher energy state of the drug in the amorphous form, providing both a dissolution and solubility enhancement of the drug [3]. In other words, an amorphous product can be brought into the supersaturated state [4], i.e., increasing the solubility above the crystalline solubility.

Since pure amorphous drugs usually are physically too unstable, it is necessary to stabilize the amorphous form using a stabilizing excipient. Hence, the formulation of drugs into amorphous solid dispersions (ASDs) has gained widespread attention over the last few decades. Such systems consist of an amorphous active pharmaceutical ingredient (API) molecularly dispersed in an excipient that acts as a matrix/carrier [5]. The role of the excipient is to prevent the amorphous API against crystallization using intermolecular interactions, anti-plasticization effects, and physical barriers to the nucleation/crystallization process [6]. Additionally, in many cases, the carriers used in ASDs can also play an important role in sustaining supersaturation during dissolution [3].

Since the introduction of ASDs, the most studied carriers in this respect use (semi-) synthetic polymers, such as hydroxypropyl methylcellulose acetate succinate (HPMCAS) or the methacrylic acid-methyl methacrylate co-polymer Eudragit^®^ L100. Despite a growing number of ASD products based on polymeric carriers reaching the market, polymeric excipients often face challenges during ASD development. For example, drug solubility in the polymeric carrier is often limited to below 30 wt% [7]. Hence, to avoid the drug’s recrystallization within the time frame of the drug product’s shelf-life, it is frequently necessary to use large portions of the selected polymeric carrier to obtain physically stable ASDs, resulting in large bulk volumes of the final dosage form and increased oral pill burden.

In recent years, preparing ASDs with the aid of proteins has been an attractive alternative to using (semi-) synthetic polymers as carriers. Proteins are composed of amino acids that offer a diverse nature of functional groups with the potential to form hydrogen bonds and electrostatic or hydrophobic interactions with API molecules. For example, gelatin has been examined as a co-former in ASDs for twelve poorly aqueous drugs [8]. In this study, all drugs could be prepared as amorphous systems with enhanced dissolution and solubility compared to the respective crystalline drugs. Furthermore, bovine serum albumin was used as a drug carrier in ASDs for dissolution enhancement compared to the crystalline drug indomethacin [9]. However, despite these favorable findings, the drug loading levels in these early studies were still comparable to those achievable with common polymeric carriers. Recently, ASDs with a high drug loading (50% *w*/*w*) have been designed for three model drugs using the protein mixture whey protein isolate (WPI) as carriers [10]. It could also be shown that drugs from these WPI-ASDs exhibited faster dissolution and higher solubility than the respective crystalline drugs.

So far, protein-based ASDs have not been directly compared to other enabling formulation approaches, such as polymer-based ASDs or nanocrystals. In this study, one protein-based ASD, two polymer-based ASDs, and one nanocrystalline formulation were prepared. Rifaximin (RFX, BCS IV), a low-solubility and low-permeability compound, was used as a model drug. RFX is a broad-spectrum antibiotic used for targeting the gastrointestinal. Clinical trials have demonstrated that RFX has poor absorption in vivo [11]. RFX ASDs formulations were studied in previous research. It has been shown that the solubility, intestinal permeability, and gastrointestinal bioavailability of RFX from ASDs improved compared to the crystal form [12,13]. RFX is marketed as tablets of 200 and 500 mg dose strength. The recommended adult dose is either 200 mg three times daily or 550 mg two times daily, depending on the indication [14]. Side effects frequently include dizziness, constipation, abdominal pain, diarrhea, flatulence, nausea, rectal tenesmus, vomiting, and pyrexia [15].

The main purpose of this study was to investigate the dissolution and solubility enhancement of a protein-based ASD using the whey protein β-lactoglobulin (BLG) and compare it to the performance of two polymer-based ASDs and one nanocrystalline formulation. BLG is the major component in WPI and is known to have hydrophobic binding sites that potentially exhibit strong binding affinities for hydrophobic compounds [16,17]. HPMCAS and Eudragit^®^ L were chosen as carriers for the ASDs based on synthetic polymers. Spray drying was used to produce the three ASDs at the weight ratio of 1:1, and the RFX nanocrystalline formulation was prepared by wet milling.

## 2. Materials and Methods

### 2.1. Materials

Rifaximin (RFX, purity ≥ 98%, molecular weight: 785.9 g/mol, pKa: 6.77) was obtained from Clarochem Ireland Ltd. (Dublin, Ireland). Lacprodan^®^ BLG Pharma Grade (BLG) was obtained from Arla Foods Ingredients (Viby, Denmark). Hydroxypropyl methylcellulose acetate succinate (HPMCAS, AQUAOT^®^, HF grade) was obtained from Shin-Etsu (Tokyo, Japan). The methacrylic acid-methyl methacrylate co-polymer Eudragit^®^ L100 (EudL) was obtained from Evonik Röhm GmbH (Darmstadt, Germany). Poloxamer 407 (Pluronic^®^ F127) and ammonium formate (≥99.995%) were obtained from Sigma-Aldrich (St. Louis, MO, USA). Methanol (HPLC grade), acetonitrile (HPLC grade), and ethanol (absolute, >99.7%) came from VWR International (Radnor, PA, USA). Water was obtained from a Millipore Milli-Q Ultra Pure water purification system (Billerica, MA, USA).

### 2.2. Preparation of the Amorphous Solid Dispersions by Spray Drying

The three ASDs at 50% drug loading (RFX: excipient = 1:1, *w*/*w*) were prepared using a Büchi B-290 spray dryer (Büchi Labortechnik AG, Flawil, Switzerland) in a closed loop configuration utilizing a dehumidifier (Büchi B-296) and an inert loop (Büchi B-295). For the BLG-ASD, BLG dissolved in water (20 mg/mL) and RFX dissolved in ethanol (20 mg/mL) were fed into the outer and inner channels of a Büchi three-fluid nozzle, respectively. A constant feed rate of 1.8 mL/min was applied. For the HPMCAS-ASD and EudL-ASD, all the compounds were dissolved in methanol at a total concentration of 20 mg/mL (10 mg/mL for RFX and excipient, respectively). The solutions were subsequently fed into the spray drier at a feed rate of 3 mL/min through a Büchi two-fluid nozzle. All the samples were spray dried under the following conditions: inlet temperature of 100 °C, atomization air flow rate of 473 L/h, drying air flow rate of ca. 35 m^3^/h, and an outlet temperature of <65 °C.

### 2.3. Preparation of the Nanocrystalline Formulation by Wet Milling

An amount of 2 g of RFX, 400 mg of poloxamer 407, and 40 g of a blend of glass beads (1.0 mm in diameter: 0.5 mm in diameter = 3:1, *w*/*w*) were weighed and transferred to a glass bottle of 200 mL. Subsequently, 40 mL of water was added to the mixture and stirred with a magnetic stir bar at 400 rpm for 24 h at room temperature. The milled RFX particles were collected and separated from the glass beads using a syringe with a 21-gauge needle and subsequently subjected to centrifugation (Microspin 12, Grant Instruments Ltd., Royston, UK) at 14.6 K rpm for 12 min. The supernatant was discarded, and the RFX particles were collected and resuspended in water by vortexing at 1000 rpm for 30 s, followed by centrifugation for 8 min. This process was repeated twice to remove poloxamer 407. The remaining RFX particles were collected and dried overnight under ambient conditions.

### 2.4. X-ray Powder Diffraction (XRPD)

XRPD diffractograms were recorded using an X’Pert PANalytical PRO X-ray diffractometer (PANalytical, Almelo, The Netherlands) with CuKα radiation (λ = 1.54187 Å). All samples were scanned in the range of 5–30° 2θ, at a scan speed of 0.067° 2θ/s and a step size of 0.026°. The acceleration voltage and current were 45 kV and 40 mA, respectively. The collected data were analyzed using X’Pert Data Viewer (version 1.2) software.

### 2.5. Modulated Differential Scanning Calorimetry (mDSC)

DSC thermograms of the prepared samples were performed using a Discovery DSC (TA instruments, New Castle, USA). A 3–5 mg sample was crimped in an aluminum Tzero pan and heated from 0 °C to 250 °C at a heating rate of 2 °C/min with an underlying modulation amplitude of 0.2120 °C and a period of 40 s. A constant flow of pure nitrogen gas with a rate of 50 mL/min was applied during the measurement (*n* = 1). The data were collected and examined with TRIOS software (TA Instruments, version 5.1.1). The glass transition temperature (T_g_, midpoint) was determined from the reversing heat flow signal.

### 2.6. Thermogravimetric Analysis (TGA)

The moisture content of the samples (*n* = 1) was measured using a Discovery TGA (TA Instruments, New Castle, DE, USA). Samples of 5–10 mg were placed in open platinum pans and heated from room temperature to 300 °C at a heating rate of 10 °C/min (*n* = 1). The weight loss (in percentage) in a temperature range between room temperature and 120 °C was determined using the TRIOS software (TA Instruments, version 5.1.1).

### 2.7. Scanning Electron Microscopy (SEM)

The morphology of the prepared samples was analyzed using an FEI Quanta™ 3D FEG (Thermo Fisher Scientific, Waltham, MA, USA). The samples were mounted on an aluminum stub with double-sided carbon tape, followed by coating with gold at a layer of 6 nm.

### 2.8. Physical Stability

The three ASD formulations were stored in desiccators either at room temperature (silica gel) or under accelerated conditions in open containers at 40 °C and 75% relative humidity (RH) containing a saturated sodium chloride suspension. Each sample was subjected to XRPD analysis after storage for 5 weeks.

### 2.9. Powder Dissolution

Powder dissolution was conducted under non-sink conditions at room temperature in three dissolution media, i.e., 0.1 M hydrochloric acid (HCl, pH 1.2), acetate buffer (pH 4.5), and fasted state simulated intestinal fluid (FaSSIF-V2, pH 6.5, Biorelevant, London, UK). All the prepared samples were sieved through a 0.125 mm sieve, and samples equivalent to 20 mg of RFX were added into an Erlenmeyer flask containing 20 mL of dissolution medium under stirring at 200 rpm. Samples of 2 mL were collected after 5, 10, 20, 40, 60, 90, and 120 min, and filtered using a 0.45 μm syringe filter (Qmax, Frisenette ApS, Knebel, Denmark). The extracted samples were immediately replaced with 2 mL of dissolution medium. The filtered samples were diluted with acetonitrile, followed by filtration using the 0.45 μm syringe filter. The powder dissolution was conducted in triplicate for each prepared formulation.

### 2.10. High-Performance Liquid Chromatography (HPLC)

The concentration of RFX was analyzed using an Agilent 1260 Infinity HPLC instrument (Agilent, Santa Clara, CA, USA) equipped with an Agilent 1290 Diode Array Detector and an Agilent column (TC-C18, 4.6 × 250 mm, 5 μm). The mobile phase consisted of 3.16 g/L of ammonium formate (pH 7.2 ± 0.05) and a methanol–acetonitrile mixture (1:1, *v*/*v*) at a volume ratio of 20 to 80. The analysis was conducted at a detection wavelength of 276 nm with a flow rate of 1.0 mL/min and an injection volume of 20 μL. The collected data were analyzed using Agilent OpenLab CDS LC 1260 software.

## 3. Results

### 3.1. Solid-State Characterization and Physical Stability

**Figure 1:**
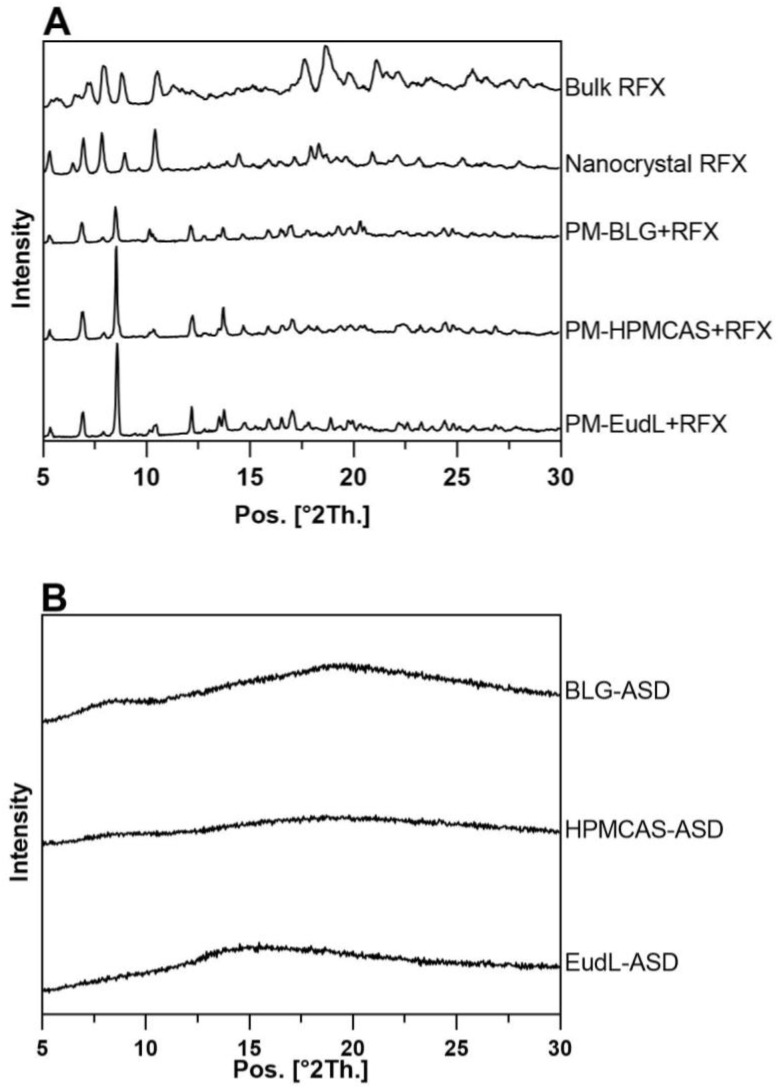

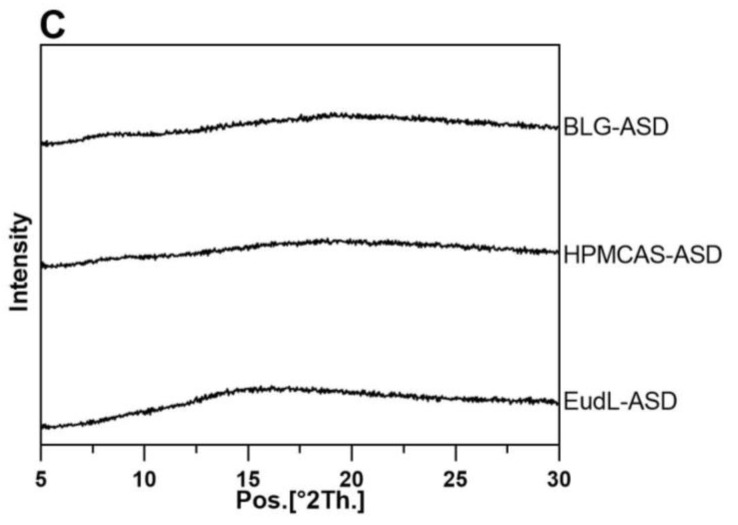
XRPD diffractograms of (**A**) bulk RFX, physical mixture (PM), (**B**) the freshly prepared ASD samples, and (**C**) the ASD samples stored for 5 weeks under accelerated conditions at 40 °C/75%RH.

The XRPD diffractograms of bulk RFX showed distinct crystalline peaks at 2θ angles of 5.7°, 7.2°, 8.9°, 10.5°, 11.8°,17.6°, and 18.7° (Figure 1A), which correspond to the peak positions and intensity reported for RFX δ-form [18]. A crystalline structure of RFX was also detected in the wet-milled RFX nanocrystals. However, with diffraction peaks at different 2θ angles (5.4°, 6.5°, 7.0°, 7.8°, 9.0°, 10.4°, 14.5°,18.0°, and 18.4°) compared to bulk RFX, suggested a polymorphic transition of RFX δ-form to the β-form polymorph [18] as a result of the wet milling process. It has previously been shown that the several polymorphic forms (α, γ, δ and ε) of RFX revert to the β-form RFX within a few hours in the presence of water [18]. On the other hand, compared to the physical mixtures (PM) (Figure 1A), the crystalline peaks of RFX were absent in the ASD samples (BLG-ASD, HPMCAS-ASD and EudL-ASD, Figure 1B). Only a diffuse halo was visible in the diffractograms, suggesting a complete amorphization of the drug. The thermal properties of RFX, the individual excipients, and the three freshly prepared ASD samples were further investigated by mDSC. As shown in Table 1, all the ASD samples revealed a single T_g_. As expected, the T_g_ of the ASDs were found in between those of pure RFX and the individual excipients. For the fresh BLG-ASD, this resulted in a slightly higher T_g_ than that of pure RFX, whereas, for the polymer-based ASDs, the T_g_ values were both lower than that of pure RFX. Hence, the BLG-ASD showed the highest T_g_ value among the three ASDs, followed by EudL-ASD and HPMCAS-ASD. The T_g_ of an amorphous system is regarded as one of the key factors influencing the physical stability of an amorphous samples. Molecular movement is reduced below the T_g_, which means the chance of molecular reorientation (as a prerequisite for nucleation and crystal growth) is decreased [19]. Upon storage for 5 weeks under accelerated conditions, all the ASD samples remained in their amorphous form and no drug crystallization was observed (Figure 1C). Furthermore, all the ASDs absorbed some moisture after storage for 5 weeks. This was most pronounced for the BLG-ASD compared to the polymeric ASDs, possibly due to a higher hygroscopicity of BLG.

### 3.2. Morphology

**Figure 2:**
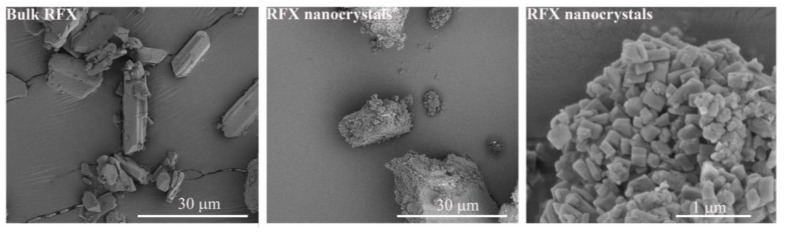
SEM images of bulk RFX (**left**) and nanocrystalline RFX ((**middle**) and (**right**)).

**Figure 3:**
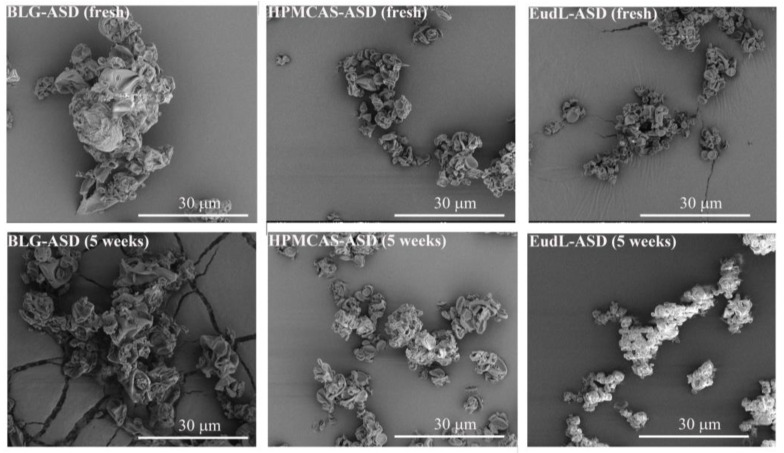
SEM images of the freshly prepared samples and the ASD samples stored for 5 weeks at accelerated conditions.

Bulk RFX showed irregular-shaped particles with a broad size distribution of 5–70 µm (Figure 2). After wet milling, the individual RFX particle size was significantly reduced to a particle size of less than 1 µm with a cubic shape. It was also observed that the nanocrystalline particles formed large agglomerates, a common phenomenon in preparing nanocrystals. One possible explanation is that most of the surfactant poloxamer 407 on the surface of nanocrystal particles was removed during resuspension and centrifugation, leading to aggregation. [20] On the other hand, BLG-ASD, HPMCAS-ASD, and EudL-ASD revealed shriveled and collapsed particles typically seen for spray-dried particles (Figure 3). The size of the BLG-ASD particles (2 to 20 µm) was slightly larger than the size of the HPMCAS-ASD (2–10 µm) and EudL-ASD (1 to 10 µm) particles on average. No significant difference in the morphology of the particles was observed in the ASD samples after preparation and upon storage for 5 weeks at accelerated conditions.

### 3.3. Powder Dissolution

The in vitro dissolution study was performed under non-sink conditions to evaluate the ability of the prepared ASDs to generate and maintain supersaturation. Considering the pH gradient in the gastrointestinal tract, dissolution profiles of the prepared formulations were investigated in three dissolution media at pH 1.2, 4.5, and 6.5 (FaSSIF-V2).

**Figure 4:**
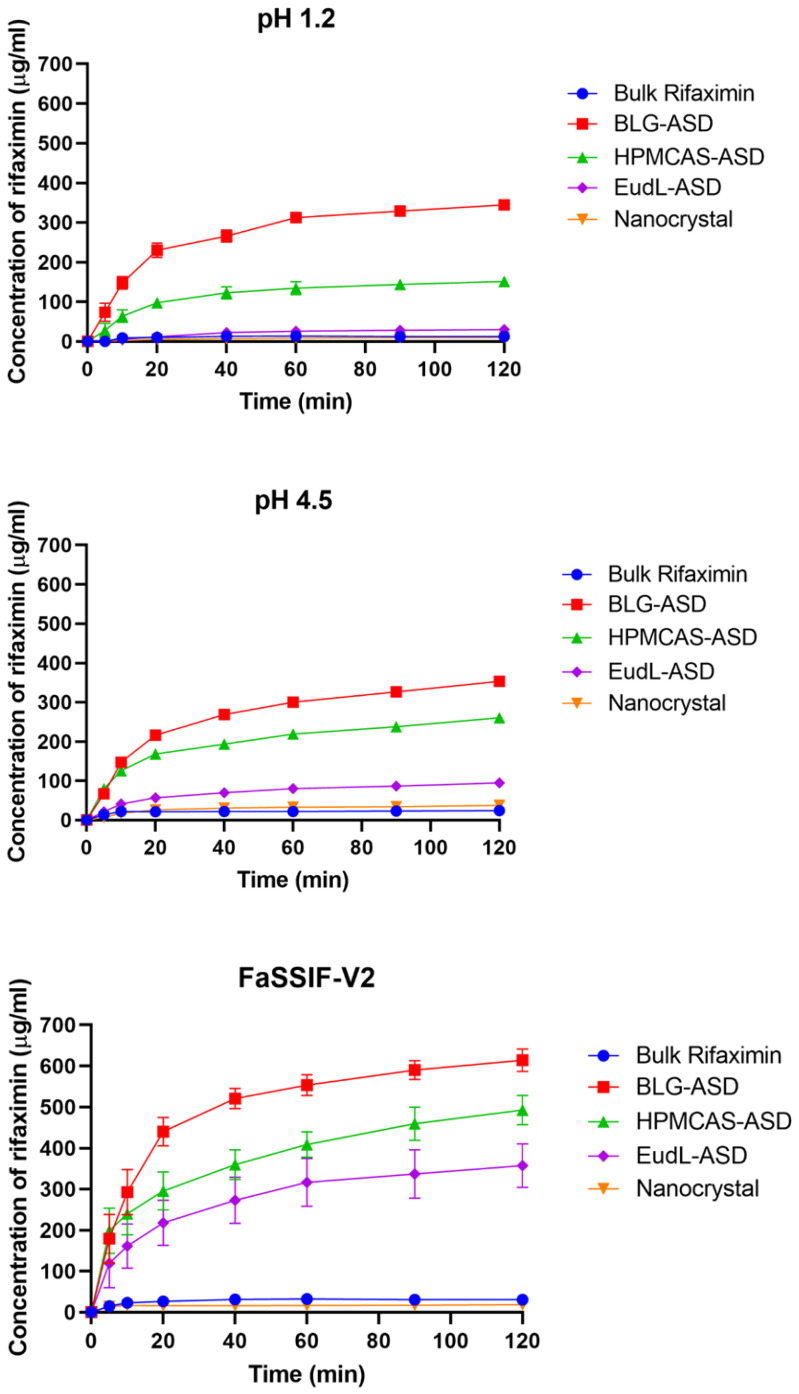
In vitro powder dissolution profiles of bulk RFX and the freshly prepared samples, in pH 1.2, pH 4.5, and FaSSIF-V2.

Bulk RFX (δ-form) exhibited a low drug release with a maximum concentration (C_max_) of 13.7 µg/mL in pH 1.2, 24.0 µg/mL in pH 4.5, and 30.3 µg/mL in pH 6.5 (Figure 4). Interestingly, the dissolution rate of the RFX nanocrystals (β-form) was not increased in any of the media, and concentration values remained below those of bulk RFX at the beginning of the experiments (<10 min). In terms of C_max_, the nanocrystals also showed lower values at pH 1.2 (C_max_: 10.3 µg/mL) and pH 6.5 (C_max_: 18.2 µg/mL), but a slightly higher value at pH 4.5 (C_max_: 37.8 µg/mL) compared to that of bulk RFX. In theory, formulations with smaller particle sizes should have an increased effective surface area and, hence, show a faster dissolution. Although the individual particle size of the nanocrystal was much smaller than the particle size of bulk RFX, the nanocrystals appeared to be agglomerated in the SEM analysis, where some of the agglomerates were larger than the particle size of RFX bulk material. Hence, the nanocrystalline formulation potentially had an apparent lower surface area from the agglomerates. In addition, it was previously shown that the β-form of RFX crystal has a lower solubility compared to δ-form RFX crystal [18]. Therefore, the change in a polymorphic structure during the wet milling process also contributed to the slower dissolution rate.

As expected, all ASDs reached higher concentrations with respect to the crystalline RFX in the three media, suggesting apparent drug supersaturation from these formulations. Furthermore, the BLG-ASD showed the fastest dissolution rate and highest solubility during the dissolution process among all the formulations. BLG-ASD exhibited a rapid dissolution rate within 20 min in the three dissolution media, following a sustained drug supersaturation. Specifically, the drug release of the BLG-ASD was similar in the media at pH 1.2 and 4.5, reaching a C_max_ of approx. 350 µg/mL at the end of the dissolution experiment. In the case of the pH 6.5 medium, the BLG-ASD had an even faster dissolution rate than the dissolution in the more acidic media (pH 1.2 and pH 4.5), reaching a C_max_ of 614.2 µg/mL at 120 min. The increased solubility of RFX could be ascribed to the surfactants in FaSSIF-V2 media, which may further contribute to the overall supersaturation capability of RFX from the BLG-ASD.

As shown in Figure 4, the release rates of the HPMCAS-ASD in the three media were lower than the release from the BLG-ASD and additionally also more pH-dependent, reaching a C_max_ of 151.3 µg/mL in pH 1.2, 260.6 µg/mL in pH 4.5 and 492.7 µg/mL in pH 6.5 at 120 min, respectively. The release rate of the EudL-ASD was even lower than those observed for the HPMCAS-ASD in all three media and also similarly pH-dependent, reaching a C_max_ of 30.2 µg/mL in pH 1.2, 94.6 µg/mL in pH 4.5 and 280.5 µg/mL in pH 6.5 at 120 min, respectively. This can be explained by the pH-dependent solubility of HPMCAS (soluble above pH 5.5) and EudL (soluble above pH 6.0) [4,21]. With the increase of pH, the enhanced solubility of the carriers would improve the dissolution behavior of RFX from these polymer-based ASD. The difference observed for the ASDs between these two polymers can potentially also result from a faster dissolution rate of the pure polymer HPMCAS compared to EudL, as previously suggested by Tze et al. [22].

As the data suggest, the carrier used in ASD plays a key role in releasing the amorphous drug and, subsequently, the degree of supersaturation obtained. In the case of pH 1.2 and 4.5, the low-solubility of the polymeric carriers HPMCAS and EudL at pH 1.2 and 4.5 reduced the performance of these ASDs compared to the BLG-ASD, and the dissolution of the drug in the HPMCAS-ASD and EudL-ASD is suggested to be mainly a result of a continuous diffusion of the amorphous drug from the insoluble carrier matrix [4,23]. The carrier BLG is soluble at all investigated pH values and could quickly dissolve or co-dissolve with the drug in the dissolution media at pH 1.2 and 4.5, resulting in a faster liberation of the amorphous drug into the dissolution medium and, consequently, an improved dissolution performance of the drug. At pH 6.5, all three carriers were soluble in this medium. However, the BLG-ASD still exhibited the fastest dissolution rate and reached the highest C_max_ values compared to the polymer-based ASDs.

## 4. Conclusions

In this study, the dissolution and solubility performance of a BLG-based ASD were investigated in three dissolution media and compared to two polymer-based ASDs and one nanocrystalline formulation. The results suggested that the BLG-based ASD outperformed the other two ASDs and the nanocrystalline formulation regarding dissolution rate and supersaturation in all three investigated media. Hence, it could be shown that BLG is a promising new carrier in developing solubility-enhancing ASD formulations.

## Figures and Tables

**Table 1:** T_g_s and moisture contents of different materials.

Materials	T_g_s (°C)	Moisture Content (%)	
		Fresh	5 Weeks
Bulk RFX	197.8		
BLG	240.4		
HPMCAS	121.6		
EudL	187.0		
BLG-ASD	200.1	3.9	5.7
HPMCAS-ASD	153.1	1.5	1.7
EudL-ASD	193.2	4.1	4.9

## Data Availability

Data can be requested from the authors.
